# Service and clinical impacts of reader bias in breast cancer screening: a retrospective study

**DOI:** 10.1093/bjr/tqad024

**Published:** 2023-12-12

**Authors:** Clarisse F de Vries, Roger T Staff, Jaroslaw A Dymiter, Moragh Boyle, Lesley A Anderson, Gerald Lip, the iCAIRD Radiology Collaboration, Corri Black, Alison D. Murray, Katie Wilde, James D Blackwood, Claire Butterly, John Zurowski, Jon Eilbeck, Colin McSkimming

**Affiliations:** Aberdeen Centre for Health Data Science, University of Aberdeen, Aberdeen AB25 2ZD, United Kingdom; Aberdeen Biomedical Imaging Centre, University of Aberdeen, Aberdeen AB25 2ZN, United Kingdom; National Health Service Grampian (NHSG), Aberdeen Royal Infirmary, Aberdeen AB25 2ZN, United Kingdom; Grampian Data Safe Haven (DaSH), University of Aberdeen, Aberdeen AB25 2ZD, United Kingdom; Aberdeen Centre for Health Data Science, University of Aberdeen, Aberdeen AB25 2ZD, United Kingdom; Aberdeen Centre for Health Data Science, University of Aberdeen, Aberdeen AB25 2ZD, United Kingdom; National Health Service Grampian (NHSG), Aberdeen Royal Infirmary, Aberdeen AB25 2ZN, United Kingdom; North East Scotland Breast Screening Centre, Aberdeen AB25 2XF, United Kingdom

**Keywords:** breast screening, mammography, reader bias, reader experience

## Abstract

**Objectives:**

To determine factors influencing reader agreement in breast screening and investigate the relationship between agreement level and patient outcomes.

**Methods:**

Reader pair agreement for 83 265 sets of mammograms from the Scottish Breast Screening service (2015-2020) was evaluated using Cohen’s kappa statistic. Each mammography examination was read by two readers, per routine screening practice, with the second initially blinded but able to choose to view the first reader’s opinion. If the two readers disagreed, a third reader arbitrated. Variation in reader agreement was examined by: whether the reader acted as the first or second reader, reader experience, and recall, cancer detection and arbitration recall rate.

**Results:**

Readers’ opinions varied by whether they acted as the first or second reader. Furthermore, reader 2 was more likely to agree with reader 1 if reader 1 was more experienced than they were, and less likely to agree if they themselves were more experienced than reader 1 (*P* < .001). Agreement was not significantly associated with cancer detection rate, overall recall rate or arbitration recall rates (*P* > .05). Lower agreement between readers led to a higher arbiter workload (*P* < .001).

**Conclusions:**

In mammography screening, the second reader’s opinion is influenced by the first reader’s opinion, with the degree of influence dependent on the readers’ relative experience levels.

**Advances in knowledge:**

While less-experienced readers relied on their more experienced reading partner, no adverse impact on service outcomes was observed. Allowing access to the first reader’s opinion may benefit newly qualified readers, but reduces independent evaluation, which may lower cancer detection rates.

## Introduction

Breast cancer is a leading cause of cancer-related death in women,^1^ and early detection considerably improves clinical outcomes.^2^ Many health systems internationally have implemented mammography-screening programmes, including the National Health Service in the United Kingdom (UK), to facilitate early detection. In the United Kingdom, women aged 50-70 years are screened by digital mammography, resulting in upwards of 2 million yearly examinations.^3^

Dual reporting of mammography, where two readers examine the mammograms to detect possible signs of cancer, with a third reader (arbiter) in case of disagreement, is the European standard.^4^ In the United States, single reading of mammograms is the norm.^5^ Women are recalled for further evaluation if an anomaly is detected on their mammograms. A 2020 review concluded that double reading in breast screening can improve outcomes relative to single reading.^6^ Relative to unblinded readers, blinded readers realize higher recall and cancer detection rates.^7–10^ The addition of arbitration to double reading improves the specificity of breast cancer screening by reducing recall rates, with minimal reduction in cancer detection rates.^11–14^ Combining blinded reading with arbitration may increase the positive predictive value of screening;^15^ however, current European guidelines do not make a recommendation on blinding.^16^ Reporting systems in the United Kingdom allow the second reader to unblind themselves to the first reader’s opinion.

Understanding potential sources of reader bias may improve breast screening service efficiency. The impact of the ability of the second reader to choose to unblind themselves is unclear. Whether any factors influence the likelihood of the second reader unblinding themselves and subsequently agreeing with the first reader is unknown. In addition, it is unclear whether either a high or low level of agreement between readers harms the outcomes of the screening service. Low levels of agreement raise questions about the ability and training of the two readers. In contrast, high levels of agreement suggest that the second reader adds limited value.

This study aims to determine factors influencing reader agreement in a medium-sized UK, dual reporting, breast screening service. The study evaluated the relationship between agreement level, arbiter workload, and patient outcomes.

## Methods

### The current system and reader training

The Scottish Breast Screening Service invites women aged 50-70 years for screening every 3 years, with women aged 71 years and older able to request a continuance of mammograms. In Scotland and the rest of the UK, mammograms are reviewed by two readers with a third arbiter (to resolve differences) to determine the need for further investigative examinations. Readers choose from four recall options: (1) Routine recall: recall in 3 years as part of the regular screening service (no abnormalities observed); (2) Technical recall: recall because of an issue with the image quality; (3) Review (symptoms): recall because the woman reported possible breast cancer symptoms at the time of mammography with a visually normal mammogram; and (4) Review required: recall because an abnormality (possible cancer) is detected on the mammogram. The second reader will review all mammograms minus technical recalls and can view the first reader’s assessment and identity. If the two readers disagree, an arbiter (with at least 2 years of experience^17^) reviews the mammograms and the previous readers' assessments to determine the final categorization. To supplement the clinical data retrieved from the screening programme, readers provided information on their years of mammogram reading experience. All readers participate in multidisciplinary team meetings and adhere to the local quality assurance process to meet the Scottish Breast Screening Programme requirements.

### Sample

Retrospective data relating to consecutive routine screening appointments (*n* = 98 829) between August 19, 2015 and March 20, 2020 at the breast screening service in the North East of Scotland (National Health Service Grampian) were obtained through the Grampian Data Safe Haven. For a standard mammography examination, two mammograms are taken of each breast: a top (craniocaudal or CC) view and a side (mediolateral oblique or MLO) view. Quality control checks excluded examinations without reader opinions and for which no valid reader information was available. Cases classified as “Technical Recall” and “Review (Symptoms)” by at least one reader were excluded from the performed statistical analyses. Finally, both readers with a low number of reads (<1000) and reader pairs with a low number of reads (<100) for the entire time period were excluded to reduce bias in the estimation of the outcome measures. Positive cancer cases were defined as cases recalled by routine screening, which were subsequently diagnosed through histology following either biopsy or surgery (a pathological biopsy score of B5 and above or post-surgical pathology classification as malignant).

### Ethics approval and data availability

The study was performed per the Declaration of Helsinki. The study received ethical approval from the Proportionate Review Sub-committee of the London—Bloomsbury Research Ethics Committee (20/LO/0563). Furthermore, the study had Public Benefit and Privacy Panel approval (1920-0258).

Access to the raw data used in this study is available on request with the appropriate permissions from the Grampian Data Safe Haven (https://www.abdn.ac.uk/iahs/facilities/grampian-data-safe-haven.php).

### Statistical analysis

Each reader pair’s agreement was quantified using Cohen’s kappa statistic.^18^ Cohen’s kappa corrects for the agreement occurring by chance. A kappa of 1 represents perfect agreement between readers. Kappa was calculated in R version 4.2.1^19^ with the “irr” package.^20^ For each reader pair, recall rates (after arbitration), cancer detection rates, and arbitration recall rates (proportion of cases the third reader decided to recall) were calculated.

The distribution of kappa was examined using the Kolmogorov-Smirnov test for normality. Paired *t*-tests were performed for each reader to compare mean kappa when they acted as reader 1 or 2 with the same corresponding partner.

Pearson correlations were performed to examine the relationship between agreement and reader experience. Pearson correlation coefficients (*r*, which ranges from −1 to 1 and measures the strength and the direction of the relationship) were calculated between agreement and (1) reader 1 year of experience, (2) reader 2 years of experience, and (3) their difference in experience (reader 2 years of experience minus reader 1 year of experience).

Linear regression was performed to evaluate the impact of reader agreement on arbiter workload. The independent variable was level of agreement and the dependent variable was the proportion of arbitrated cases.

Pearson correlations were also performed to examine the relationships between agreement and recall rate, cancer detection rate, and arbitration recall rate.

All statistical analyses were performed using R (version 4.2.1). A *P*-value <.05 was considered statistically significant.

## Results

**Figure 1.**
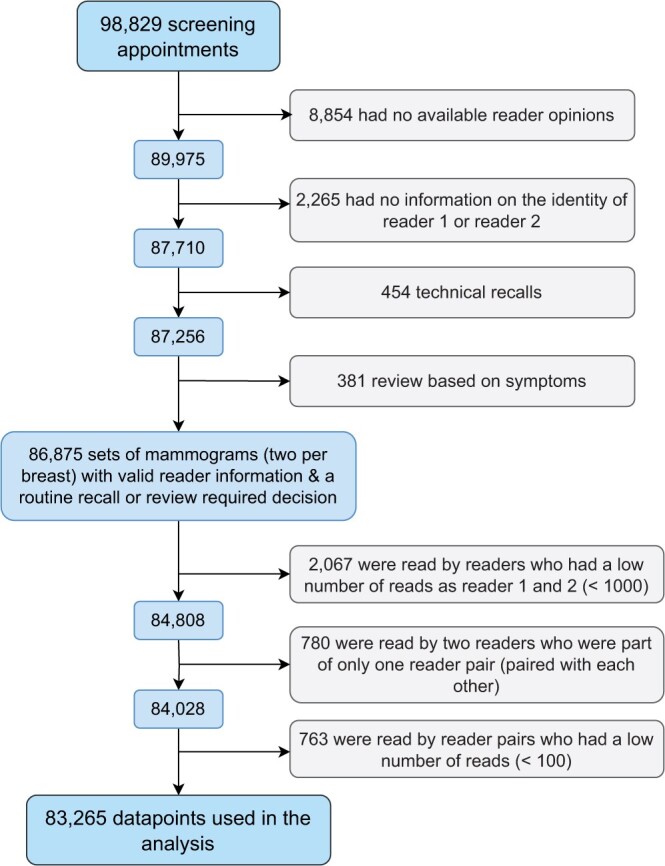
Flow diagram of included and excluded datapoints.

Figure 1 shows a flow diagram of the included and excluded data. There were 11 readers (81 reader pairs) for the 83 265 cases (sets of mammograms) evaluated. On average, readers had 11 (range 1-20) years of experience. Of the 83 265 cases evaluated, 3642 (4.4%) were recalled, and 712 cancers (8.6 per 1000) were detected. The age-range of the women included in the study was 50-92 years.

**Figure 2.**
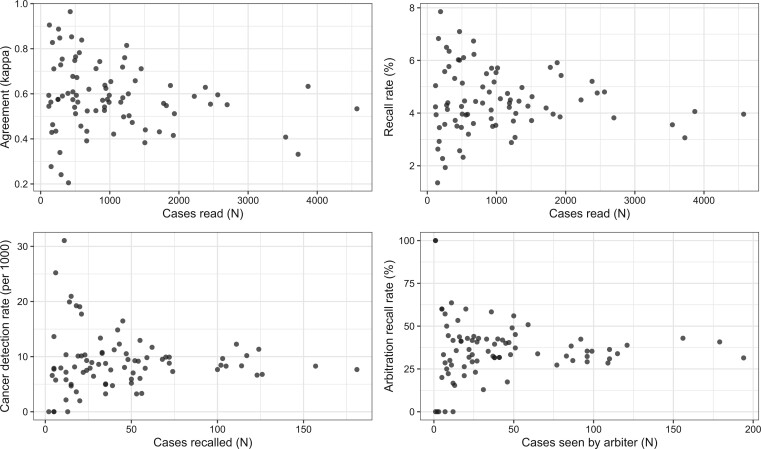
Scatterplots of agreement, recall rate, cancer detection rate, and arbitration recall rate for each reader pair versus the number of cases read, recalled, or seen by the arbiter for that pair.

Figure 2 shows descriptive scatterplots of agreement, recall rate, cancer detection rate, and arbitration recall rate for each reader pair plotted against the number of cases (*N*) read, recalled, or seen by the arbiter for that pair. Each plot broadly shows the same pattern with a larger range at lower *N* and fewer data points at higher *N* (showing that fewer reader pairs read larger volumes). Agreement was normally distributed, with a mean of 0.581 (standard error 0.017). The number of cases read by each reader pair ranged from 118 to 4573. For each pair, agreement varied from 0.21 to 0.96, recall rates from 1.4% to 7.9%, cancer detection rates from 0 to 31.1 per 1000 and arbitration recall rates from 0% to 100%.

**Figure 3.**
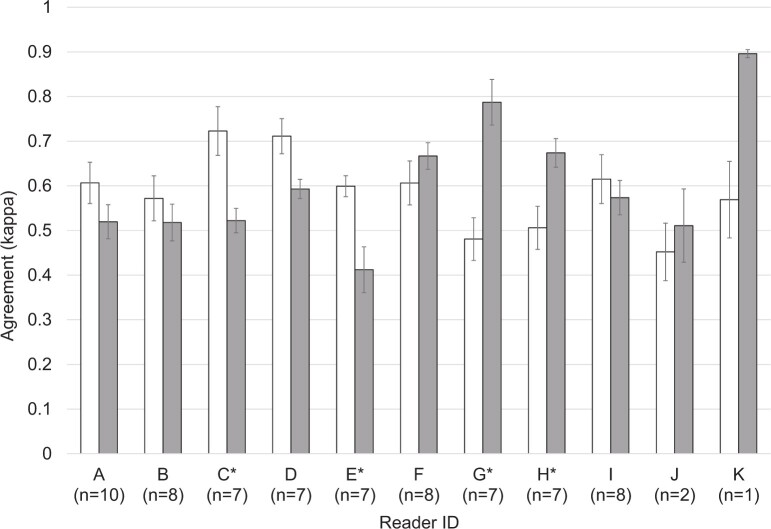
Mean agreement for each reader as the first reader (white) and as the second reader (grey). The error bars represent standard errors. The numbers in brackets on the x-axis indicate the number of paired data points. Asterisks (*) indicate readers who show a significant difference in agreement between their role as first or second reader (paired *t*-test *P* < .05). A paired *t*-test could not be performed for reader K because there was only one paired data point.

Figure 3 shows the mean agreement for each reader acting as either the first (white column) or second reader (grey column). Agreement was significantly different for readers C, E, G, and H (paired *t*-tests; *P*_C_ = .024, *P*_E_ = .039, *P*_G_ = .006 and *P*_H_ = .005) when they acted as the first or second reader. Agreement for the remaining readers did not significantly differ with their role as first or second reader (*P*_A_ = .291, *P*_B_ = .792, *P*_D_ = .054, *P*_F_ = .419, *P*_I_ = .772, *P*_J_ = .873). A paired *t*-test could not be performed for reader K because there was only one paired data point.

**Figure 4.**
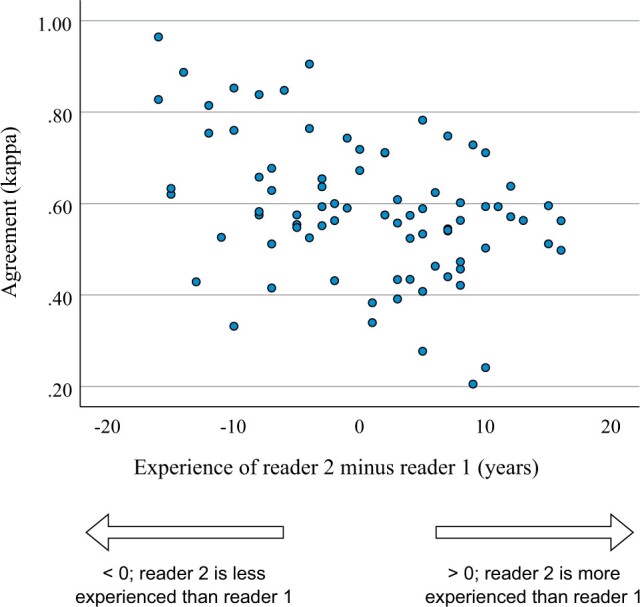
Scatterplot of agreement for each reader pair versus the difference in the readers’ experience (reader 2 minus reader 1 years of experience).

Reader agreement was positively associated with reader 1 experience (*r* = 0.330, *P* = .003) and negatively associated with reader 2 experience (*r* = −0.235, *P* = .035). In addition, agreement was also associated with the difference in reader experience (reader 2 minus reader 1 experience; *r* = −0.395, *P* < .001) (Figure 4). These results show that reader 2 is more likely to agree with reader 1 if: (1) reader 1 was experienced; (2) reader 2 was inexperienced; and (3) reader 2 was inexperienced relative to reader 1.

Agreement was negatively associated with the proportion of arbitrated cases (linear regression; *P* < 0.001). For every 0.1 increase in agreement, the percentage of arbitrated cases decreased by 0.9%. For example, a reader pair with an agreement of 0.8 would send 36 per 1000 fewer cases to the arbiter compared to a reader pair with an agreement of 0.4.

Agreement was not significantly associated with recall rates (*P* = .352), cancer detection rates (*P* = .9999), or arbitration recall rates (*P* = .606).

## Discussion

This study shows that in the UK’s dual reading breast screening service, the agreement between readers depends on their relative experience and the order in which they read the mammograms. Agreement is higher when the first reader is more experienced than the second reader, and lower when the second reader is more experienced than the first reader. While these results indicate an absence of fully independent reading, no adverse impact on service outcomes was observed. Experienced readers, therefore, play an important role in maintaining service outcomes within acceptable standards.

A 2020 study examining first reader performance in a cohort of 1 million mammography examinations concluded, consistent with these findings, that significant variation existed in reader opinion.^21^ A retrospective study of multiple sites has also demonstrated non-independent reporting in second readers unblinded to the first reader’s opinion.^15^ Relative to blinded readers, unblinded second readers were more likely to recall if the first reader chose to recall. Our study supports and extends these findings by showing that the first reader’s degree of influence on the second reader depends on their relative experience levels. This would suggest that reader experience levels could be used in service design to optimize performance. For example, newly qualified readers could be introduced to the service as unblinded second readers to provide them with an additional learning opportunity.

The main strength of our study is the provenance of the consecutively acquired data as part of the Scottish Breast Screening Service. The service is well-established and fully computerized, allowing direct data capture. The study is limited to readers in one North East of Scotland screening service. However, as part of the Scottish services quality assurance programme, the centre’s performance is frequently reviewed and found to meet the Scottish breast screening standards.^22^ Other limitations include the lack of detail regarding the individual readers’ ability, history, behaviour, and standing. Since this was a retrospective study, it was impossible to determine whether the second reader chose to unblind themselves to the opinion of the first reader. However, the high agreement observed when a second reader has less experience than the first suggests that the second reader does not independently form their opinion.

Furthermore, this study does not rule out the existence of an association between agreement and patient outcomes. Estimates of the outcome measures reported in this study showed large variability for reader pairs that had read fewer sets of mammograms. Non-linear associations may also exist. In addition, the study did not include interval cancer (IC) data (cancers diagnosed between screening cycles) as the number of ICs for each reader pair would be too small to be meaningfully used for statistical analysis.

The future of breast screening could see artificial intelligence (AI) systems implemented in clinical practice. Unblinding human readers to an AI reader will likely affect their opinions. AI has the potential to support the screening service and help detect additional cancers.^23^ However, a recent study showed that breast screening radiologists across experience levels are susceptible to being influenced by an incorrect AI opinion, with inexperienced readers most impacted.^24^ As with “human-to-human” interactions considered here, “AI-to-human” interactions should be a subject of future research if the full benefit of AI is to be realized.

## Conclusion

There is a clear pattern of association between readers’ experience and the agreement between them in a dual reading breast screening service, with less-experienced readers likely to agree with their more experienced reading partners. Understanding the interaction between readers of different experience levels is vital to optimizing the breast screening service and ensuring that inexperienced readers are provided with training opportunities.
